# In vivo 3D measurement of moxifloxacin and gatifloxacin distributions in the mouse cornea using multiphoton microscopy

**DOI:** 10.1038/srep25339

**Published:** 2016-05-03

**Authors:** Seunghun Lee, Jun Ho Lee, Jin Hyoung Park, Yeoreum Yoon, Wan Kyun Chung, Hungwon Tchah, Myoung Joon Kim, Ki Hean Kim

**Affiliations:** 1 Department of Mechanical Engineering, Pohang University of Science and Technology, 77 Cheongam-ro, Nam-gu, Pohang, Gyeoungbuk 37673, Rep. of Korea; 2 Department of Ophthalmology, University of Ulsan College of Medicine, Asan Medical Center, 88 Olympic-ro 43-gil, Songpa-gu, Seoul 05505, Rep. of Korea; 3 Division of Integrative Biosciences and Biotechnology, Pohang University of Science and Technology, 77 Cheongam-ro, Nam-gu, Pohang, Gyeoungbuk 37673, Rep. of Korea

## Abstract

Moxifloxacin and gatifloxacin are fourth-generation fluoroquinolone antibiotics used in the clinic to prevent or treat ocular infections. Their pharmacokinetics in the cornea is usually measured from extracted ocular fluids or tissues, and *in vivo* direct measurement is difficult. In this study multiphoton microscopy (MPM), which is a 3D optical microscopic technique based on multiphoton fluorescence, was applied to the measurement of moxifloxacin and gatifloxacin distribution in the cornea. Intrinsic multiphoton fluorescence properties of moxifloxacin and gatifloxacin were characterized, and their distributions in mouse cornea *in vivo* were measured by 3D MPM imaging. Both moxifloxacin and gatifloxacin had similar multiphoton spectra, while moxifloxacin had stronger fluorescence than gatifloxacin. MPM imaging of mouse cornea *in vivo* showed (1) moxifloxacin had good penetration through the superficial corneal epithelium, while gatifloxacin had relatively poor penetration, (2) both ophthalmic solutions had high intracellular distribution. *In vivo* MPM results were consistent with previous studies. This study demonstrates the feasibility of MPM as a method for *in vivo* direct measurement of moxifloxacin and gatifloxacin in the cornea.

Moxifloxacin and gatifloxacin are fourth-generation fluoroquinolones, widely used to prevent or treat ocular infection. They have high potency against both gram-positive and gram-negative bacterial pathogens. As ophthalmic solutions, pharmacokinetic properties such as diffusion rate across the cornea and time duration of minimum inhibitory concentration are important^1^. Ocular pharmacokinetics of moxifloxacin and gatifloxacin have been analyzed by measuring their concentration in the tear film, cornea, aqueous humor, and vitreous humor of the eye using either high performance liquid chromatography (HPLC) with fluorescence detection^1^,^2^,^3^ or liquid chromatography with tandem mass spectrometry (LC/MS)^4^,^5^. HPLC with fluorescence detection utilizes intrinsic fluorescence property of moxifloxacin and gatifloxacin. Since these methods require sampling of ocular fluids or tissues, *in vivo* measurement in the cornea is difficult. Furthermore, spatial information in tissue is not available due to homogenization during sample preparation.

Optical microscopy for measuring moxifloxacin and gatifloxacin in the cornea will be useful by providing information of their spatial distribution via imaging. *In vivo* repetitive measurement will be possible, and longitudinal imaging will allow to monitor temporal changes of their distribution. Therefore detail pharmacokinetic study and sample variation measurement would be possible. Various optical microscopic techniques such as confocal microscopy (CM) and multiphoton microscopy (MPM) have been used for measuring molecule distribution in the cornea^6^,^7^. CM is a 3D laser scanning microscopic technique based on fluorescence, where fluorophores are excited by absorption of single energetic excitation photon. Usually molecules are conjugated with fluorescent dyes such as fluorescein for CM imaging, unless they have intrinsic fluorescence. CM has been used to visualize corneal distribution of riboflavin based on its intrinsic fluorescence^8^. Both moxifloxacin and gatifloxacin have intrinsic fluorescence, but their excitation spectra are in the range deep ultra-violet wavelength^9^,^10^. Therefore, CM is not appropriate for *in vivo* imaging of moxifloxacin and gatifloxacin due to phototoxicity. MPM is a 3D fluorescence microscopic technique based on multiphoton excitation (MPE) of fluorophores. MPE is a nonlinear fluorescence process, in which fluorophores are excited by simultaneously absorbing two or more excitation photons, each having insufficient energy for excitation transition^11^,^12^. MPM uses longer excitation wavelengths than do typical fluorescence methods including CM. Therefore, MPM may be applicable to *in vivo* measurement of moxifloxacin and gatifloxacin by using near infrared excitation wavelengths, if they have intrinsic multiphoton fluorescence. MPM has 3D resolutions due to confinement of MPE at the focal volume of excitation light as nonlinear effect.

In this report, we verified and characterized multiphoton fluorescence of moxifloxacin and gatifloxacin in their ophthalmic solutions as well as aqueous solutions by using MPM. *In vivo* 3D distributions of moxifloxacin and gatifloxacin in the mouse cornea were measured by MPM imaging after topical administration. Temporal changes of their distribution were measured by longitudinal MPM imaging for 2 hours after administration.

## Results

### Multiphoton fluorescence characteristics of moxifloxacin and gatifloxacin

**Figure 1:**
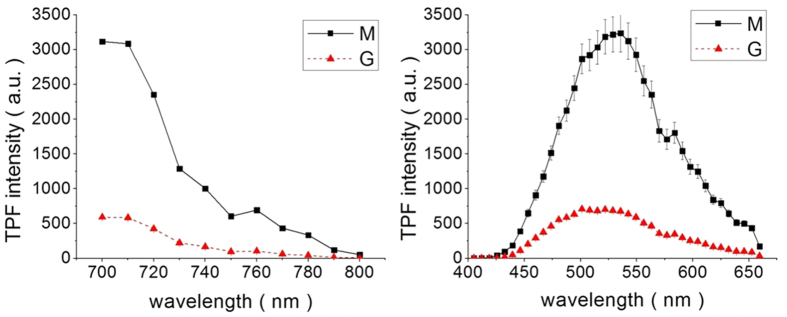
Excitation and emission spectra of moxifloxacin and gatifloxacin ophthalmic solution samples measured by MPM. (**a**) excitation spectrum, (**b**) emission spectrum. M and G indicate moxifloxacin ophthalmic solution and gatifloxacin ophthalmic solution, respectively.

Multiphoton fluorescence properties of both moxifloxacin and gatifloxacin ophthalmic solutions were measured by MPM imaging, and their excitation and emission spectra are shown in Fig. 1(a,b), respectively. These spectra were normalized by the solution concentration because moxifloxacin and gatifloxacin ophthalmic solutions had different concentrations: 0.5% and 0.3% for moxifloxacin and gatifloxacin, respectively. In the measurement of emission spectra, 790 nm excitation wavelength was used. Excitation and emission spectra of the two ophthalmic solutions showed that moxifloxacin has approximately 5–10 times stronger fluorescence than gatifloxacin. Other than the difference in fluorescence intensity, both excitation and emission spectra of the two ophthalmic solutions are similar in shape. Excitation spectra of both ophthalmic solutions have spectral ranges from 700 nm to 800 nm and have maximum intensities at 700 nm. Excitation spectra shorter than 700 nm could not be measured because of leakage of the excitation source in the current microscope setup and the limitation of laser tuning range. Emission spectra of both ophthalmic solutions have spectral ranges from 450 nm to 660 nm and have maximum intensities between 500 nm and 550 nm. In order to verify that fluorescence of these ophthalmic solutions comes from their active pharmaceutical ingredients (APIs), aqueous solutions of moxifloxacin hydrochloride and gatifloxacin, APIs of the two ophthalmic solutions, were prepared and multiphoton spectra were measured. Aqueous solutions of the two APIs showed similar excitation and emission spectra as those of corresponding ophthalmic solutions (See Supplementary Fig. S1). Aqueous solutions of the two APIs showed slightly different fluorescence intensities compared to their corresponding ophthalmic solutions. Fluorescence intensities of moxifloxacin hydrochloride and gatifloxacin aqueous solutions were approximately 1.4 times and 0.93 times of those of corresponding ophthalmic solutions.

Fluorescence intensity as a function of excitation power was measured and results showed that fluorescence intensity was proportional to 1.89 power and 1.73 power of excitation power in cases of moxifloxacin and gatifloxacin ophthalmic solutions, respectively. Since these power values were close to 2, it was concluded that their intrinsic fluorescence was two-photon fluorescence process. There was variation in fluorescence intensity of ophthalmic solution bottles, although shapes of excitation and emission spectra were consistent. Quantification of the fluorescence intensity for the concentration measurement may require pre-calibration of the ophthalmic solution bottle. The excitation and emission spectra shown in Fig. 1 were obtained by averaging results of multiple experiments.

### Spatial distribution of moxifloxacin and gatifloxacin in the mouse cornea *in vivo*

**Figure 2:**
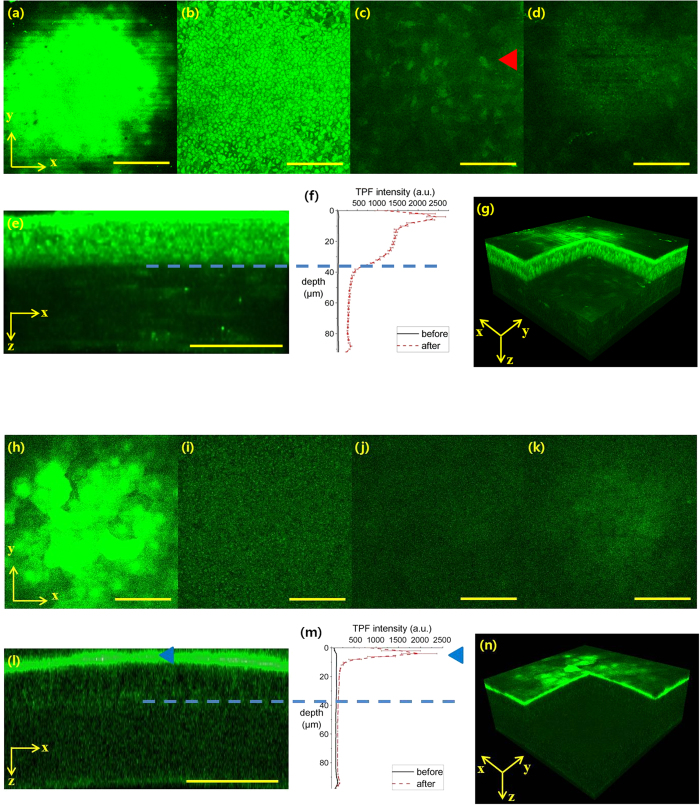
*In vivo* MPM images and average fluorescence intensity depth profiles of moxifloxacin and gatifloxacin administered mouse corneas in 15 min after administration. (**a–d**) MPM frontal images of the moxifloxacin administered mouse cornea in four corneal layers of the superficial epithelium (**a**), basal epithelium (**b**), stroma (**c**), and endothelium (**d**). (**e,f**) an MPM cross-sectional image of the moxifloxacin administered mouse cornea and the average intensity profile with depth. (**g**) 3D reconstructed MPM image of the moxifloxacin administrated cornea. (**h–k**) MPM frontal images of the gatifloxacin administered mouse cornea in four corneal layers of the superficial epithelium (**h**), basal epithelium (**i**), stroma (**j**), and endothelium (**k**). (**l,m**) an MPM cross-sectional image of the gatifloxacin administered mouse cornea and the average intensity profile with depth. (**n**) 3D reconstructed MPM image of the gatifloxacin administrated cornea. A red arrow head marks a keratocyte, a blue arrow head marks the superficial epithelium, and blue dotted lines indicate the boundary between the epithelium and stroma. Scale bars are 100 μm.

3D distribution of moxifloxacin and gatifloxacin in the mouse cornea was visualized by MPM imaging in 15 min after topical administration, and results are shown in Fig. 2 (See Supplementary videos S1 and S2). MPM images and fluorescence intensity profiles of moxifloxacin and gatifloxacin administered mouse corneas are shown in Fig. 2(a–g,h–n), respectively. For each ophthalmic solution, MPM frontal images at 4 different depths of the superficial epithelium, basal epithelium, stroma, and endothelium, a MPM cross-sectional image, average fluorescence intensity profiles across the cornea, and a 3D rendered MPM image are shown. MPM frontal images of the moxifloxacin administered mouse cornea show strong fluorescence in the superficial and basal epithelia, and relatively weak fluorescence in the stroma and endothelium. Cells in the cornea including epithelial cells in the epithelium, keratocytes in the stroma (red arrowhead), and endothelial cells in the endothelium, are visible in MPM images with stronger intracellular fluorescence than outside. MPM cross-sectional image shows fluorescence intensity distribution within the cornea clearly. There is a band of strong fluorescence in the superficial epithelium indicating moxifloxacin on the surface. Below the superficial epithelium, individual epithelial cells are visible and strong fluorescence is maintained across the epithelium. The stroma has much weaker fluorescence than the epithelium and shows sparsely distributed keratocytes. The endothelium appears as a thin and weak fluorescent band. The average intensity profiles with depth before and after moxifloxacin administration show only autofluorescence, and combination of autofluorescence and moxifloxacin fluorescence in the cornea, respectively. Autofluorescence in the cornea was measured by using the same excitation power as the one used for the imaging after moxifloxacin administration. Since autofluorescence is significantly smaller compared to moxifloxacin fluorescence, it is concluded that fluorescence in the cornea after moxifloxacin administration is mostly moxifloxacin fluorescence. Depth profile of average fluorescence in the cornea after moxifloxacin administration shows maximum intensity on the corneal surface (or in the superficial epithelium) and then intensity decreases with depth, partly as the effect of topical administration and barrier function of the superficial epithelium. Fluorescence in the epithelium below the surface is still strong, approximately 57% of that on the surface, and decreases gradually with depth down to the basal epithelium. Fluorescence in the stroma drops to approximately 12% of that in the superficial epithelium and is relatively uniform across the stroma layer. Fluorescence in the endothelium is slightly stronger than that in the stroma.

Fluorescence distribution of the gatifloxacin administered mouse cornea is quite different from that of the moxifloxacin administered mouse cornea. MPM frontal images of 4 different corneal layers and a MPM cross-sectional image show strong fluorescence in the superficial epithelium only and weak fluorescence in the other lower corneal layers, indicating much less penetration of gatifloxacin in the cornea compared to moxifloxacin. Like moxifloxacin, MPM images of the gatifloxacin treated cornea show stronger fluorescence inside the cells than outside. The depth profile of average fluorescence intensity in the gatifloxacin treated cornea shows maximum intensity on the corneal surface (or in the superficial epithelium) marked with a blue arrow head and low intensities close to the level of autofluorescence in the lower corneal layers. Autofluorescence of the cornea before gatifloxacin administration is stronger than that of moxifloxacin case by using higher excitation power for MPM imaging. Therefore, fluorescence intensity profile of the gatifloxacin administered mouse cornea shows almost no penetration of gatifloxacin in 15 min after topical administration.

### Temporal changes of moxifloxacin and gatifloxacin in the mouse cornea *in vivo*

**Figure 3:**
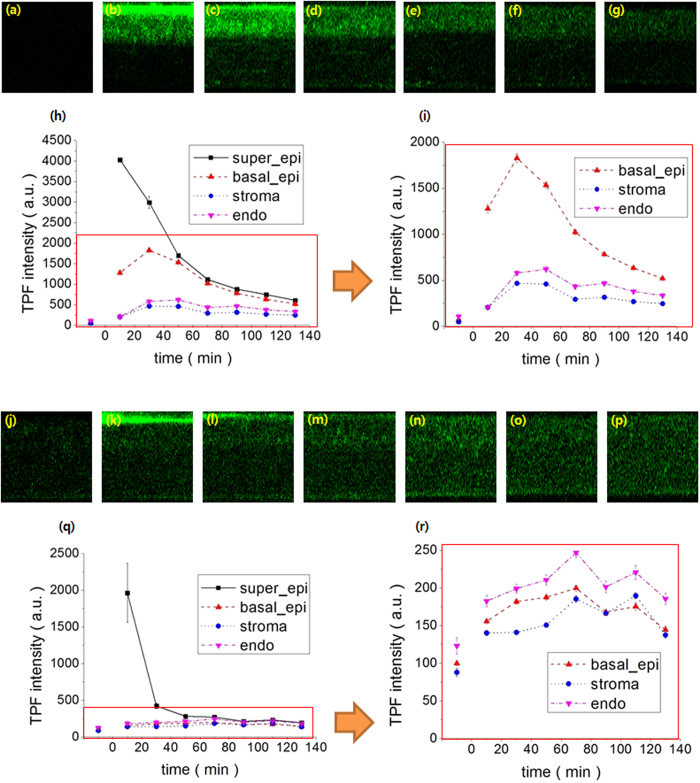
Longitudinal *in vivo* cross-sectional MPM images of moxifloxacin and gatifloxacin administered mouse corneas in the x-z plane and temporal fluorescence intensity profiles in four different corneal layers for 2 hours after administration. (**a–g**) MPM images of moxifloxacin administered mouse corneas before administration, 10, 30, 50, 70, 90, and 110 min after administration. (**h–l**) temporal profiles of average intensities in the superficial epithelium, basal epithelium, stroma, and endothelium of the moxifloxacin administered cornea and magnified temporal intensity profiles in the lower three corneal layers. (**j–p**) MPM images of gatifloxacin administered mouse corneas before administration, 10, 30, 50, 70, 90, and 110 min after administration. (**q**,**r**) temporal profiles of average intensities in the superficial epithelium, basal epithelium, stroma, and endothelium of the gatifloxacin administered cornea and magnified temporal intensity profiles in the lower three corneal layers.

Temporal changes of moxifloxacin and gatifloxacin distribution in the mouse cornea after topical administration were measured by longitudinal MPM imaging for approximately two hours after administration, and results are shown in Fig. 3. MPM cross-sectional images of moxifloxacin and gatifloxacin administered mouse corneas before administration and several time points after administration from 10 min to 110 min with 20 min interval are shown in Fig. 3(a–g,j–p) respectively. Temporal profiles of average fluorescence intensities in 4 different layers of moxifloxacin and gatifloxacin administered corneas are shown in Fig. 3(h,i,q,r), respectively. *In vivo* longitudinal MPM images and temporal intensity profiles of the moxifloxacin administered cornea show (1) before administration, the cornea has weak autofluorescence, (2) in 10 min after administration, the superficial and basal epitheliums have strong fluorescence, and the stroma and endothelium have weak fluorescence to the level of autofluorescence. Fluorescence intensities in the basal epithelium and stroma/endothelium are approximately 30% and 4% of that in the superficial epithelium respectively, (3) in 30 min after administration, the superficial epithelium shows fluorescence decrease, and the other layers of basal epithelium, stroma, and endothelium show fluorescence increases. Fluorescence intensities in the basal epithelium and stroma/endothelium are approximately 45% and 13% of the maximum intensity in the superficial epithelium in 10 min after administration, (4) in 50 min after and onward, all the corneal layers show gradual decreases of fluorescence, (5) in 130 min, the basal epithelium and stroma/endothelium have fluorescence intensities approximately 2–3 times stronger than the autofluorescence levels. Temporal intensity profiles of the four corneal layers show that (1) the superficial epithelium has a monotonic intensity decrease with time, (2) the basal epithelium has an intensity increase until 30 min after administration and a decrease later on, (3) the stroma and endothelium show intensity increases until 30 min and 50 min after administration, and then slow decreases.

*In vivo* longitudinal MPM images of the gatifloxacin administered cornea show (1) before administration, the cornea has some autofluorescence, (2) in 10 min after administration, the superficial epithelium has strong fluorescence from gatifloxacin but the other layers have low fluorescence intensities similar to the levels of autofluorescence, (3) in 30–70 min after administration, the superficial epithelium has a fluorescence decrease and the other layers have gradual increases. The endothelium becomes brighter with time, and its maximum intensity is approximately 7% of the maximum intensity in the superficial epithelium in 10 min after administration and twice of autofluorescence, (4) in 90 min and onward, all the corneal layers have relatively constant intensities within approximately twice the autofluorescence levels. The temporal intensity profiles of the four corneal layers show (1) the superficial epithelium has the maximum fluorescence intensity in 10 min after administration, and a large intensity drop in 30 min, and relatively constant intensities from 50 min and later, (2) the basal epithelium has a low intensity, slightly stronger than that of autofluorescence, in 10 min after adminsteration. Its intensity has increases until 70 min, and a slow intensity decrease later on, (3) the stroma and endothelium has similar intensity patterns as that of basal epithelium.

## Discussion

MPM was applied to the measurement of moxifloxacin and gatifloxacin distribution in the mouse cornea *in vivo* by 3D imaging based on their intrinsic multiphoton fluorescence. Multiphoton fluorescence of moxifloxacin and gatifloxacin ophthalmic solutions was characterized by measuring their excitation and emission spectra. NIR excitation light from 700 nm to 800 nm could excite these ophthalmic solutions, and the fluorescence process turned out to be two-photon fluorescence by measuring a quadratic relationship between fluorescence intensity and excitation power. MPM was applied to *in vivo* measurement of moxifloxacin and gatifloxacin distribution in the mouse cornea after topical administration. Fluorescence of topically administered ophthalmic solutions was stronger than intrinsic autofluorescence of the untreated cornea, and their distributions in cornea were visualized clearly by MPM imaging. MPM imaging of the mouse cornea after topical administration showed that moxifloxacin penetrates the cornea better than gatifloxacin, consistent with previous pharmacokinetic studies with extracted ocular fluid. Higher lipophilicity and aqueous solubility of moxifloxacin allows better penetration across the cornea than gatifloxacin^1^,^13^. MPM imaging of both moxifloxacin and gatifloxacin administered corneas visualized cells in the cornea due to their high intracellular concentration. Longitudinal MPM imaging of the mouse cornea after topical administration showed temporal changes of moxifloxacin and gatifloxacin distribution in four different corneal layers.

MPM imaging of both moxifloxacin and gatifloxacin administrated corneas showed maximum fluorescence intensities in the superficial epithelium, lower fluorescence intensities in the stroma and endothelium, and almost negligible intensities in the aqueous humor. Maximum fluorescence intensities in the superficial epithelium confirmed the roles of the superficial epithelium layer as both barrier and reservoir for ophthalmic solutions^14^. Stroma and endothelium had stronger fluorescence compared to the aqueous humor partially due to higher concentration of moxifloxacin and gatifloxacin inside corneal cells than the outside. Likewise, dense cell distribution in the endothelium made higher fluorescence intensities than those in the stroma.

Fluorescence intensities in the aqueous humor were very low at the current imaging condition. It is important to measure drug penetration into the aqueous humor in case of prophylaxis of postoperative endophthalmitis. Although the current study focused on drug distribution in the cornea, sensitive measurement in the aqueous humor is possible in principle. It is because MPM is highly sensitive and there is no noise source such as autofluorescence in the aqueous humor. MPM sensitivity was measured by using diluted moxifloxacin ophthalmic solution samples and the same excitation power used for the *in vivo* imaging was used in this measurement. Minimum detectable moxifloxacin concentration was approximately 1.93 × 10^−5^%, which is lower than that in the aqueous humor in previous studies^1^,^5^.

The excitation wavelength used for imaging the mouse cornea was 790 nm. Since the wavelength for maximum excitation was shorter than 700 nm, the current excitation wavelength was not optimal. However, excitation wavelength of 790 nm was chosen in order to minimize autofluorescence in the cornea. Most autofluorescence in the cornea comes from nicotinamide adenine dinucleotide (phosphate) (NAD(P)H), flavins, retinoids, lipofuscin, and elastin^15^ and this excitation becomes stronger with shorter excitation wavelengths^16^. Further investigation will be needed to find the optimal excitation wavelength, balancing between excitation efficiency and autofluorescence background noise in corneal imaging.

MPM imaging of the mouse cornea after topical administration was performed three times for both moxifloxacin and gatifloxacin, and the results were consistent each other. However, there was fluorescence intensity variation among experiments, which might be partly due to sample variation. There was also fluorescence intensity variation among ophthalmic solution bottles, and this variation might come from composition difference of the ophthalmic solutions and photo-bleaching effect during storage. Therefore, quantitative measurement of ophthalmic solution concentration in the cornea would require prior fluorescence intensity calibration of the ophthalmic solution.

All the *in vivo* studies were performed by using the mouse cornea, whose thickness is approximately 100 μm. Therefore, the study results may not be directly applicable to large animal corneas whose thicknesses can be 600–700 μm. Therefore, the next step will be to make this technique applicable to larger animal corneas such as those of rabbits.

*In vivo* MPM images of the mouse cornea showed that both moxifloxacin and gatifloxacin were concentrated inside the cells (See Supplementary Fig. S2). This indicates that these ophthalmic solutions may be used as contrast agents for labeling cells and other microorganisms in cornea or other tissues. Since they are drugs approved for human use, this may open the possibility of using them in clinical applications as a contrast agent.

Although MPM imaging could measure moxifloxacin and gatifloxacin distribution in the mouse cornea *in vivo* by 3D imaging, this method has several limitations. 1) Visualization of the two fluoroquinolone ophthalmic solutions was based on their intrinsic multiphoton fluorescence. Therefore, this method is not applicable to other non-fluorescent drugs. 2) Although MPM enabled visualization of moxifloxacin and gatifloxacin distribution at the cellular level, the imaging field of view was limited to a few hundred micrometers in length. 3) MPM based method may not be able to perform quantitative measurements in the opaque cornea because the excitation laser will be attenuated during propagation through the cornea due to light scattering and the generated fluorescence will be attenuated accordingly. Therefore, MPM based method will be applicable to normal transparent cornea. 4) Current MPM based measurement in the *in vivo* mouse cornea required mouse anesthesia during imaging due to slow imaging speed. Since anesthesia changes fluid dynamics on the ocular surface due to decreased tear production and the loss of blinking, corneal absorption of anesthetized mouse cornea can be different from that of unanesthetized mouse cornea^17^,^18^. We tried to avoid this artifact by closing the eyelid 30 seconds after administration and changing immersion media every 10 minutes to mimic blinking. High speed MPM methods will be needed for measurement without anesthesia^19^,^20^.

In conclusion, MPM was applied to characterize intrinsic multiphoton fluorescence of the two fluoroquinolone ophthalmic solutions. MPM imaging of the *in vivo* mouse cornea after topical administration directly visualized their distribution at the cellular level, and longitudinal MPM imaging of the same mouse cornea showed temporal changes of their distribution in the corneal layers. This study demonstrates the feasibility of MPM as a method for *in vivo* direct measurement of moxifloxacin and gatifloxacin in the cornea.

## Methods

### Multiphoton microscopy system

A Leica SP5 multiphoton microscope system equipped with a Titanium Sapphire laser (Chameleon vision, Coherent) was used for MPM imaging. Excitation wavelength was tunable from 680 nm to 1080 nm. Emission light could be spectrally resolved up to 4 channels by using combinations of dichroic mirrors and emission filters. Measurement of the emission spectrum was also possible by spectrally dispersing emission light with a prism and collecting with a moving slit in the system. A 25× objective lens (HCX IRAPO L 25 × 0.95 NA water immersion, Leica) was used. Images in the x-y plane consisting of 512 × 512 pixels were typically acquired, and the field of view (FOV) was 310 μm × 310 μm. 3D imaging was performed by acquiring multiple x-y plane images with stepwise translation in the z direction.

### Reagents

Vigamox (Alcon Laboratories Inc., Fort worth, US) and Gatiflo (Handock Inc., Eun-Seong, Korea), which are moxifloxacin 0.5% and gatifloxacin 0.3% ophthalmic solutions respectively, were used in both the characterization of intrinsic multiphoton fluorescence and MPM imaging of the mouse cornea. In order to verify that multiphoton fluorescence properties of their ophthalmic solutions come from the active pharmaceutical ingredients (APIs), moxifloxacin hydrochloride (sc-205758, Santa Cruz Biotechnology Inc., Dallas, US) and gatifloxacin (sc-204762, Santa Cruz Biotechnology Inc., Dallas, US), were also used in the characterization.

### Multiphoton fluorescence characterization

Stock ophthalmic solutions of moxifloxacin (0.5%) and gatifloxacin (0.3%) were used without dilution. Moxifloxacin hydrochloride and gatifloxacin were dissolved in distilled water at the same concentrations as the ophthalmic solution samples. Since the pH of moxifloxacin and gatilfoxacin ophthalmic solutions were 6.8 and 5.8, respectively, the pH values of their aqueous solutions were adjusted to 7 and 5.8 by adding hydrochloride and sodium hydroxide. 50 μL of the solutions was placed in a well slide, coverslipped, and sealed with nail polish. The solution samples were imaged with the excitation power of approximately 6 mW at the sample and the imaging speed of 0.4 frames/s. Average intensities were calculated from the image. Excitation spectra were measured by changing the excitation wavelength from 700 nm to 800 nm with a step size of 10 nm. Emission spectra were measured from 400 nm to 660 nm with a step size of 7 nm. Excitation wavelength in the emission spectrum measurement was set at 790 nm. Fluorescence intensity variation of the solution sample as a function of excitation power was measured in order to characterize the fluorescence process. The solution samples were prepared by diluting stock ophthalmic solutions to approximately 10–20% by mixing with phosphate buffered saline. Fluorescence intensities were measured with at least 5 different excitation power values, and the relationship between them was analyzed by linear curve fitting in log scale.

### *In vivo* multiphoton microscope imaging of mouse cornea

For *in vivo* imaging of the mouse cornea, Balb/c female mice aged 5–6 weeks were used. These mice were bred at the animal facility of POSTECH Biotech Center under specific pathogen free conditions. The animal experiment method was approved by POSTECH’s Institutional Animal Care and Use Committee (IACUC, approval number POSTECH-2015-0030-C1). All experiments were done in accordance with the approved guidelines. For *in vivo* MPM imaging of mouse corneas, mice were anesthetized with a mixture of isoflurane and oxygen gases during imaging. Body temperature was maintained at 37 °C with heating pads embedded in the eye holder. Ophthalmic solution of 10μL was topically administered on the left eye, and the eyelid was closed for 30 seconds. A mouse eye holder was used to fix the mouse eye during MPM imaging. A detailed description of the mouse eye holder and surgical process was published elsewhere^21^,^22^. A viscous liquid (GenTeal Gel, Novartis) was applied to the eyes as immersion media for the objective lens.

Spatial distributions of moxifloxacin and gatifloxacin in the mouse corneas were measured by performing MPM imaging in 15 min after topical administration. Excitation wavelength was set at 790 nm for both moxifloxacin and gatifloxacin. This excitation wavelength was chosen to minimize background noise due to autofluorescence in the mouse cornea. Excitation power was approximately 10.4 mW and 30.8 mW at the samples for moxifloxacin and gatifloxacin, respectively. Emission light was collected in a single channel by using a long-pass filter and a short-pass filter (455–680 nm). Imaging speed was 0.2 frames/s, and approximately 50 frames in the x-y plane were acquired for 3D imaging with a step size of 2 μm in the z direction. Prior to topical administration, MPM imaging was performed based on autofluorescence as a control. Then, MPM imaging was repeated on the same mouse cornea after topical administration. Temporal changes of moxifloxacin and gatifloxacin distributions in the mouse cornea were measured by performing MPM imaging periodically after topical administration. The imaging speed and excitation power were 0.8 frames/s, 15.2 mW, and 0.2 frames/s, 30.8 mW for moxifloxacin and gatifloxacin, respectively. Imaging was performed before administration as a control, and then every 20 min from 10 min to 130 min after administration. All other conditions were the same as those for the spatial distribution measurements. These MPM imaging experiments of the mouse cornea was performed at least 3 times for each case in order to find the repeatability, and total 15 mice were used.

### Analysis of *in vivo* multiphoton microscope images

The Amira software package was used for 3D reconstruction. 3D MPM images were analyzed to calculate depth profiles of the average fluorescence intensity. Since the mouse cornea is curved in shape, a small region of the 3D MPM image, which was relatively flat, was selected as the region of interest (ROI). The ROI was approximately 129 μm × 129 μm in the x-y plane. This ROI was sub-divided into 7 × 7 sub-blocks and average intensity values were calculated in the individual blocks (See Supplementary Fig. S3). The average intensity and standard deviation at each depth were calculated from the average intensity values of the sub-blocks.

## Additional Information

**How to cite this article**: Lee, S. *et al*. In vivo 3D measurement of moxifloxacin and gatifloxacin distributions in the mouse cornea using multiphoton microscopy. *Sci. Rep*. **6**, 25339; doi: 10.1038/srep25339 (2016).

## Supplementary Material

Supplementary Information

Supplementary Video S1

Supplementary Video S2
