# Supplementary material for: In vivo 3D measurement of moxifloxacin and gatifloxacin distributions in the mouse cornea using multiphoton microscopy
Source: Sci Rep. 2016 May 3;6:25339. doi: 10.1038/srep25339 (PMC4853790; doi:10.1038/srep25339)
Supplement: Supplementary Information [file srep25339-s1.pdf]

In vivo 3D measurement of moxifloxacin and gatifloxacin distributions in the mouse cornea using multiphoton microscopy

Seunghun Lee[1,a], Jun Ho Lee[1,a], Jin Hyoung Park[3], Yeoreum Yoon[1], Wan Kyun Chung[1], Hungwon Tchah[3], Ki Hean Kim[1,2,*], Myoung Joon Kim[3,*]

[1] Department of Mechanical Engineering, Pohang University of Science and Technology, 77 Cheongam-ro, Nam-gu, Pohang , Gyeoungbuk 37673, Rep. of Korea

[2] Division of Integrative Biosciences and Biotechnology, Pohang University of Science and Technology, 77 Cheongam-ro, Nam-gu, Pohang , Gyeoungbuk 37673, Rep. of Korea

[3] Department of Ophthalmology, University of Ulsan College of Medicine, Asan Medical Center, 88 Olympic-ro 43-gil, Songpa-gu, Seoul 05505, Rep. of Korea

[a]: equal contribution *: corresponding authors

Table of contents

Supplementary figure S1 - Emission spectra of moxifloxacin and gatifloxacin in their aqueous solutions and ophthalmic solutions.

Supplementary figure S2. MPM images of superficial epithelium after 15min after moxifloxacin and gatifloxacin administration

Supplementary figure S3 - Analysis of average fluorescence intensity in the MPM cornea images.

Supplementary figure legends

Supplementary figure S1. Emission spectra of moxifloxacin and gatifloxacin in their aqueous solutions and ophthalmic solutions. (a) emission spectra of 0.5 % moxifloxacin ophthalmic solution and 0.5 % moxifloxacin hydrochloride aqueous solution. (b) emission spectra of 0.3 % gatifloxacin ophthalmic solution and 0.3% gatifloxacin aqueous solution.

Supplementary figure S2. MPM images of superficial epithelium after 15min after moxifloxacin and gatifloxacin administration. (a) superficial epithelium of moxifloxacin administrated cornea. (b) superficial epithelium of gatifloxacin administrated cornea

Supplementary figure S3. Analysis of average fluorescence intensity in the MPM cornea images. (a) region of interest (ROI) (129 μm x 129 μm) in the epithelium image. (b) ROI (129 μm x 129 μm) in the endothelium image. (c) 7x7 sub-blocks in the ROI of (a).

Supplementary figures

Supplementary figure S1.

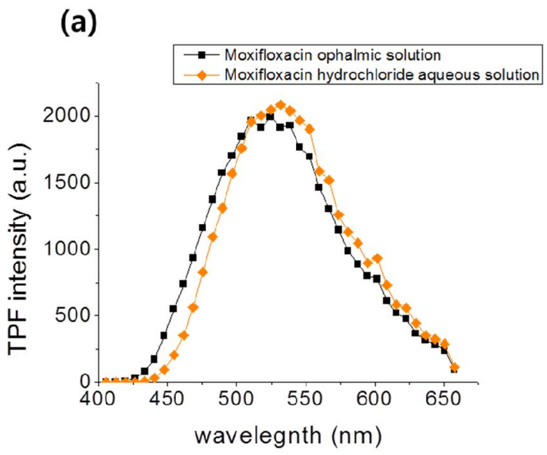

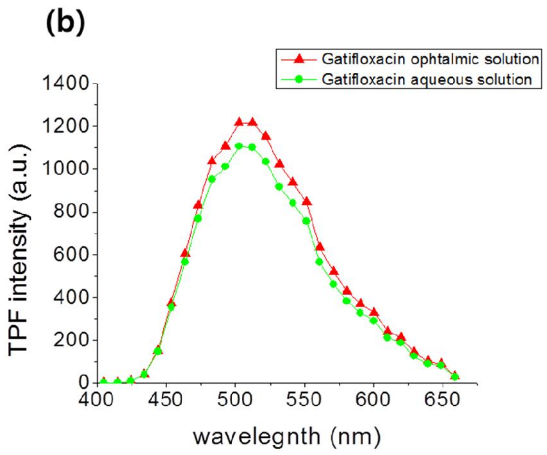

Supplementary figure S2.

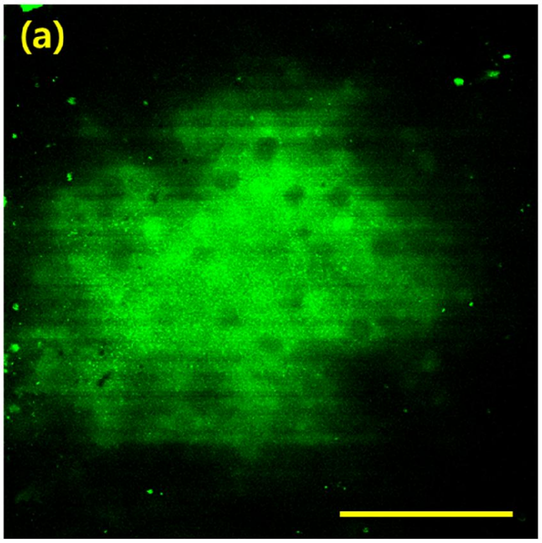

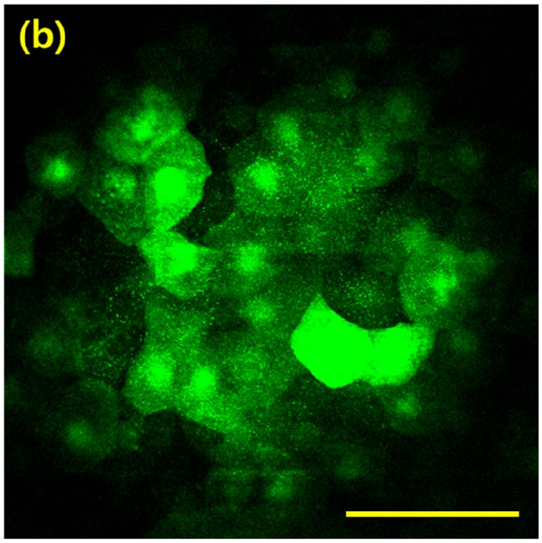

Supplementary figure S3.

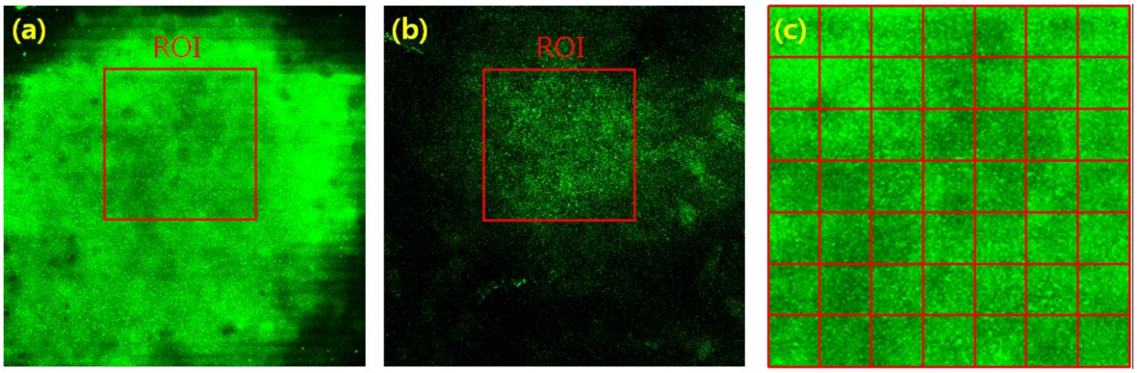

Supplementary video legends

Supplementary video S1: a 3D multiphoton image of the moxifloxacin treated mouse cornea in 15 min after topical administration.

Supplementary video S2: a 3D multiphoton image of the gatifloxacin treated mouse cornea in 15 min after topical administration.
